# Validity Evaluation of the College Student Physical Literacy Questionnaire

**DOI:** 10.3389/fpubh.2022.856659

**Published:** 2022-05-26

**Authors:** Lin Luo, Naiqing Song, Jin Huang, Xiaodong Zou, Junfeng Yuan, Chenli Li, Jie Yang, Ling Zhou, Liping Zhang, Shiyan Luo, Xin Gao

**Affiliations:** ^1^East China Normal University-Xuhui Postdoctoral Workstation, Shanghai, China; ^2^College of Physical Education, Guizhou Normal University, Guiyang, China; ^3^Basic Education Research Center, Southwest University, Chongqing, China; ^4^College of Exercise and Health, Shandong Institute of Physical Education, Rizhao, China

**Keywords:** physical literacy, college students, questionnaire, reliability, validity

## Abstract

**Background:**

Physical literacy (PL) is an important tool to promote physical activity of individuals, and the level of physical literacy of individuals affects their physical activity behaviors. Currently, the physical fitness of college students in China is a prominent issue, and assessing physical literacy among college students may provide tools and directions to further promote physical fitness and precisely intervene in physical activity behaviors of college students in the future. This study aimed to develop a college student physical literacy questionnaire (CSPLQ) to address the lack of currently available physical literacy assessment tools for Chinese college students. We hoped to collect validity evidence of this questionnaire to measure the validity of the physical literacy self-assessment questionnaire among Chinese university students.

**Methods:**

An initial pool of items was obtained from existing research instruments, literature, and expert advice. An expert review panel evaluated its content. A subsequent validation process reduced the pool of items. We conducted a validation factor analysis of the CSPLQ using structural equation modeling. The relationship between physical literacy and other variables was also examined using correlation analysis.

**Results:**

The item content validity index (ICVI) of CSPLQ was 0.70–0.95. The CSPLQ was composed of a total of 38 items across 3 domains (physical and behavioral domain, affective domain, and cognitive domain) and 7 dimensions (motor skills, motor skills, physical activity, perceptions of healthy living, perceptions of physical activity, motivation to engage in physical activity, and confidence to engage in physical activity). The factor validity of the CSPLQ was determined by significant loading of all items on their expected factors, with good data model fit and good stability between two independent samples were demonstrated. Each subscale had a Cronbach α coefficient >0.9 and was strongly correlated with each other. The correlation coefficients between college students' physical literacy and other variables, including athletic ability, physical condition, physical attractiveness, physical fitness, frequency of physical activity, and length of physical activity, all reached a significance level of *P* < 0.05.

**Conclusion:**

The CSPLQ has sufficient evidence of validity. The development of the instrument showed evidence of validity for the content, response process, internal structure, and relationships with other variables.

## Introduction

Physical literacy (PL) refers to “the motivation, confidence, physical ability, knowledge and understanding to value and actively participate in physical activity (1).” Physical literacy is the ability of an individual to achieve a healthy lifestyle (2), and is very important for the development of an individual's physical and mental health (3). In 2015, the International Physical Literacy Association (IPLA) issued a consensus statement on physical literacy (4), which was supported by more than 1,300 sports organization leaders and experts. The IPLA considers physical literacy to have four interconnected and essential elements: motivation and confidence (emotional domain), physical ability (physical domain), knowledge and understanding (cognitive domain), and behavioral participation in lifelong physical activity (behavioral domain) (4). Individuals with higher levels of physical literacy will be more confident and capable of participating in various physical activities (5), while individuals with lower levels of physical literacy will have less physical activity behaviors (6).

Taking physical literacy as an important means to promote individual physical activity, it is necessary to use physical literacy assessment tools to help understand people's physical literacy level (7). In the past two decades, scholars in different countries have developed some physical literacy assessment tools or assessment models. Such as Canadian Agility and Movement Skill Assessment (CAMSA) (8), physical literacy assessment for youth tools (PLAY) (7), Canadian assessment of physical literac (CAPL) (9), Portuguese Physical Literacy Assessment Questionnaire (15–18 years old), Australia's physical literacy assessment model based on Structure of the observed learning outcome (SOLO) (10). However, because different assessment tools are based on different conceptual models, they are applicable to different ages and populations. For example, the three physical literacy assessment tools in Canada (CAMSA, PLAY, CAPL) and Portugal's (PPLA-Q) are designed according to the stage and continuous characteristics of children and adolescents' growth and development (8, 9, 11). CAPL is suitable for 8–12 years old, PLAY is suitable for 7–12 years old, CAMSA is suitable for 8–12 years old, PPLA-Q is suitable for 15–18 years old. Australia has established an assessment model based on the “SOLO classification theory” to observe the performance results of various elements of physical literacy in specific situations, which can be applied to all groups of people, but there is still no specific quantitative assessment tool. Although some scholars have proposed that developing individual physical literacy in early life is more conducive to participation in sports and physical activities throughout life, the structural model of physical literacy should not be limited to children and early adolescents, and more ages and groups should be explored to achieve People maintain purposeful physical pursuits and activities throughout life (12).

In China, the physical health of students has attracted the attention of the government. According to the latest national student physical fitness test data, college students are the group with the largest number of students in all academic stages whose physical fitness test scores do not meet the national test requirements (13). Under this realistic background, the physical literacy assessment of college students may provide tools and directions for further promoting the physical health of college students and accurately intervening in the physical health of college students in the future. To our knowledge of published articles, there are currently few tools available for assessing physical literacy in college students.

Zhao (14) constructed a structural model of adolescent physical literacy evaluation (14) from four dimensions of sports knowledge, sports habits, sports conditions, and sports spirit. However, this evaluation model is quite different from the IPLA's definition of the conceptual model of physical literacy. Ma et al. (15) used the Perceived Physical Literacy Instrument (PPLI) for validation among Chinese college students (15). Although this study showed that PPLI had better construct validity and reliability in 622 Guangdong college students, whether PPLI adaptation for more Chinese college students remains to be further explored. First of all, the modeling population of PPLI is professional physical education teachers, and its evaluation content is very professional in sports. Secondly, PPLI uses Cantonese, and its language habits are quite different from Mandarin, which is currently mainly used in China. The validation samples used in this study were also from Cantonese-speaking regions. In addition, in the assessment tools of physical literacy of children and adolescents, most motor skills tests are used to reflect the physical ability of individuals, such as PLAY and CAPL. However, some scholars believe that more motor skills tests may not be conducive to large-scale assessment of physical literacy. On this basis, they proposed that the use of self-reported motor skill levels may facilitate the development of large-scale physical literacy assessments (12).

Therefore, in order to meet the needs of large-scale physical literacy assessment of Chinese college students, we aimed to design a preliminary questionnaire to identify the physical literacy of Chinese college students (College student physical literacy questionnaire, CSPLQ). Then collect the validity evidence of the questionnaire to measure its validity in the physical literacy assessment of Chinese college students, so as to objectify and quantify the subjective and qualitative college students' physical literacy.

## Materials and Methods

### Research Process

**Figure 1:**
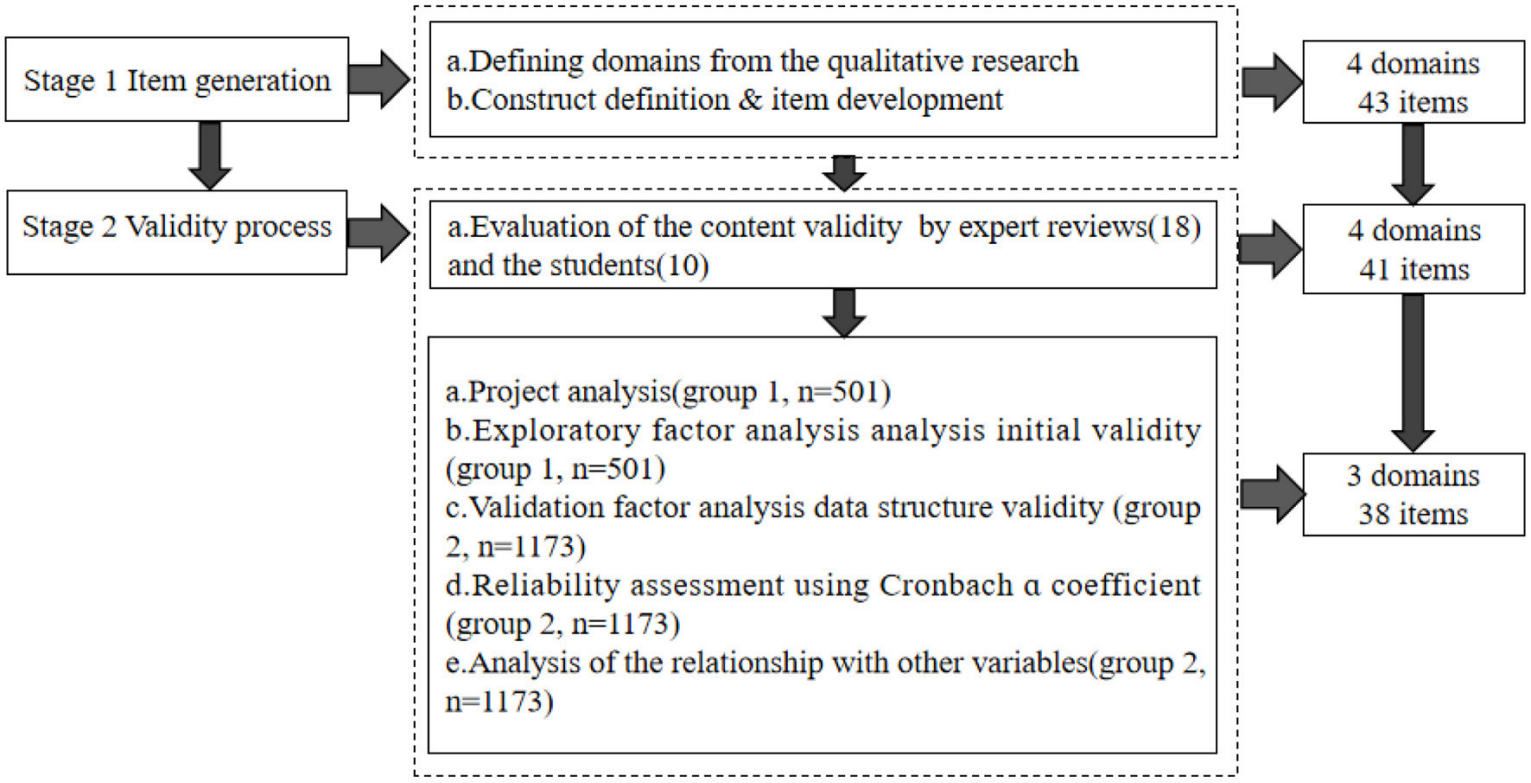
The CSPLQ research process.

The research process of the CSPLQ consists of two stages: item generation and validity process (Figure 1). Referring to the validity framework recommended by AERA-APA (1999), this study uses content, response process, internal structure, and relations with other variables to measure the validity process of CSPLQ (16, 17).

### Item Generation

The research team evaluated existing literature on physical literacy assessments and compared scholars' views on the concept of physical literacy. In the end, we chose Chinese scholar Li et al. to define the concept of “physical literacy” (18). According to her concept, physical literacy is divided into four fields, including emotional field (motivation and confidence), physical field (physical ability), cognitive domain (knowledge and understanding), behavior domain (behavior of participation in lifelong physical activity). Entries in each field of inclusion must be persistent and suitable for self-reporting. All secondary and tertiary indicators in the initial indicators were designed with the help of an expert group consisting of three experts and scholars with more than 10 years of experience in sports measurement and evaluation. These items were rated using a five-point Likert scale, with a score of 1 = completely disagree to 5 = completely agree.

### Validity Process

To determine the effectiveness of the content, we invited our second panel of experts. This group comes from our literature search from CNKI and obtained 18 Chinese scholars (8 with senior titles, 5 with associate senior titles, 2 associate researchers, and 3 postdoctoral fellows) with relevant research. To align experts with their concepts of content effectiveness metrics (relevance, clarity, comprehensiveness), we explained the definitions of these metrics to experts. Relevance is defined as the ability of design questions to reflect content. Clarity is clarity with regard to wording and description of concepts. Finally, a questionnaire that includes all content areas is defined as comprehensive. We emailed them the original question. The subjects of the questionnaire are divided into objective questions and subjective questions. The objective questions are in the form of a Likert scale, and the evaluation level is “1 = not at all important ~ 5 very important,” and the importance of the first-level indicators, second-level indicators and observation points (third-level indicators) is investigated. The higher the score, the higher the recognition of the reasonableness of the indicator by the surveyed experts. Subjective questions mainly seek experts' suggestions on whether the indicators are reasonable, increase or decrease, and corrections. After gathering expert input, the initial three-person panel revised some questions based on the feedback. The next step was to evaluate the descriptions of the questionnaire with the help of 10 college student volunteers. They completed questionnaires and gave their suggestions for difficulties understanding the descriptions of the questions or answers. We have rephrased the items that needed revision to be grammatically and colloquially acceptable and understandable. We sent the corrected questionnaire back to the second-round panelists, asking them to indicate their level of agreement on the relevance and clarity of each item and the comprehensiveness of the questionnaire. They were asked to rate the clarity and reliability of each item and the comprehensiveness of the questionnaire on a scale from 1 to 5 (1 = completely unreasonable to 5 = very reasonable), collect expert answers, and calculate content validity metrics. At this stage, items were retained if the Item Content Validity Index (ICVI) was ≥0.70 (19), indicating acceptable agreement. The IRA for relevance and clarity of the new questionnaire was estimated using the Scale Content Validity Index (SCVI). To estimate SCVI, we averaged S-CVI/Ave by summarizing ICVI and dividing by the number of items. The comprehensiveness of the questionnaire is described using the total number of experts. This process questionnaire went from 43 items to 41 items. The questionnaire is prepared according to the language habit of Mandarin Chinese and can be completed in 10–15 min. For the assessment of the internal structure, we used data from two questionnaire surveys (groups 1 and 2). For the evaluation of construct validity, two questionnaires (Group 1 and Group 2) were used. Item analysis followed by exploratory factor analysis (EFA) was conducted for Group 1. Item redundancy was determined based on the following assumptions: (a) loading factor > 0.4 for each item, (b) mean inter-item correlation > 0.20, and (c) no overlap or wording redundancy between items (19). This process turned the questionnaire into 38 items in 3 domains. Questionnaire validation was performed by validated factor analysis (CFA) using Group 2 data to assess dimensions as a measure of the internal structure of the questionnaire (20). The dimensions of the instrument were assessed using selected fit index criteria. The criteria used were: (a) Root mean squared error approximation (RMSEA) < 0.1 (21); (b) *p*-values should be significant and chi-square divided by degrees of freedom < 3 (22); and (c) Comparative fit index (CFI) > 0.90 (23). After model fitting, the internal consistency of the questionnaire was measured using Cronbach's alpha for the total questionnaire and the three sub-questionnaires (24). The relationship between exercise capacity, physical condition, physical attractiveness, physical fitness, frequency of physical activity and time spent in physical activity was analyzed using correlation analysis. All questionnaires were administered between 15 March 2021 and 10 May 2021. The questionnaire was administered on a 5-point Likert scale, 1 = completely inconsistent to 5 = completely consistent. Statistical analysis of all data was performed using SPSS 22.0 software.

### Participant Recruitment

In order to study the content structure of the questionnaire, we conducted two data collections in total. The pre-test survey site is selected in the university town of the researcher's city. The pre-test selected college students from 7 colleges and universities at the 211 level, provincial key, ordinary second, third, and junior college levels. The electronic questionnaires were distributed through the “Mike” questionnaire platform, and a total of 501 valid questionnaires were returned (meeting the requirement of 5–10 times the number of questions). Among them, 238 were boys (47.50%), with an average age of 19.88 ± 1.21 years. There were 263 girls (52.50%), with an average age of 19.71 ± 1.13 years. There are 362 (72.26%) college students with rural household registration and 139 (27.74%) urban household registration students. The formal test randomly selected 15 colleges and universities in the eastern, western, southern, northern and central regions of China. For the formal survey, a total of 15 colleges and universities in the eastern, western, southern, northern, and central regions of China were selected for random sampling. A total of 1,217 questionnaires were received, and a total of 1,173 valid questionnaires were received. Among them, there were 533 boys (45.44%), with an average age of 19.98 ± 1.40 years. There were 640 girls (54.56%), with an average age of 19.09 ± 1.47 years. There are 911 students with rural household registration (77.66%) and 262 students with urban household registration (22.34%).

## Results

### Item Generation

Based on the relevant literature on physical literacy assessment and the recommendations of a three-person expert group, we divided college students' physical literacy into four first-level indicators, including emotional, physical, cognitive, and behavioral domains. For these four first-level indicators, we have expanded the second-level and third-level indicators. The first evaluation index system of this study was obtained (Table 1).

**Table 1:** The first evaluation index system.

**First-level indicator**	**Secondary indicators**	**Three-level indicator**
A1 Physical domain	B1 Motor skills	Basic movement skills
		Core stability
		Motor coordination
		Action accuracy
		Hand-eye coordination
	B2 Motion skills	Body rhythm
		Speed quality
		Strength quality
		Endurance quality
		Flexibility
		Agility
		Balance
		Athletic ability
		Motor learning ability
A2 Behavioral domain	B3 Physical activity	Daily physical activity
		Moderate-intensity physical activity time per week
		Frequency of physical activity during the week
	B4 Sedentary behavior	Daily screen behavior
		Daily sedentary behavior
		Daily lying time
A3 Cognitive domain	B5 Cognition of healthy lifestyle	Daily diet
		Sleep
		Living habit
		Health
	B6 Cognition of physical activity	Physiological responses to exercise
		Physical activity safety
		Physical activity principles
		Principle of sedentary behavior
A4 Emotional domain	B7 Motivation to participate in physical activity	Value judgement
		Emotional needs
		Body type needs
		Social needs
		Health needs
		Physical examination needs
		School rules
		Friend influence
		Parents urge
		Interest driven
		Aesthetic preference
		Activity appreciation
	B8 Confidence to participate in physical life	Confidence in bodily functioning
		Confidence in mobility
		Confidence in body shape

### Validity Process

#### Expert Review and Response Process

A preliminary questionnaire with 43 questions was designed according to the first version of the questionnaire index system, and then the number of items in the questionnaire was reduced to 41 after validity analysis. Among them, the two observation points of “daily lying time” and “frequency of physical activity in a week” have been deleted, and two observation points have been modified, such as “body shape preference” being changed to “aesthetic preference,” and “activity participation” being changed to “activity” appreciation. “The ICVI of the last questionnaire ranged from 0.70 to 0.95. Indicates that the majority of experts agree with the selected item and its related issues. The consistency of the relevance, clarity and comprehensiveness scores of the final 41-item questionnaires were 82.33, 78.99 and 81.02%.

Ten college student volunteers helped evaluate the descriptions of the questionnaire. After evaluation, the description of Q24 “I have mastered the knowledge of sports safety protection” was changed to “I have mastered the knowledge of sports safety protection,” and the Q36 “I like watching various sports events” was changed to “I like watching various sports activities very much (competition).”

#### Internal Structural Analysis

##### Project Analysis

SPSS 22.0 software was used to analyze the basic characteristics of the measurement items on the 501 survey data in the pre-test. Table 2 provides the mean, standard deviation, skewness, and kurtosis of the 41 questions answered in the initial questionnaire on physical literacy of college students. From the skewness and kurtosis analysis results of the 41 items, their absolute values are all <2 (19), indicating that the respondents' responses to the items belong to a normal distribution. In order to further analyze the degree of distinction of the items, the survey data were divided into high and low groups of 25% up and down according to the total score of the questionnaire. A *t*-test was performed on the two groups of data to compare the differences between the high and low groups on each item (19). The analysis results are shown in Table 2 for the CR values. Except for Q17 which did not reach the significant level of 0.05, the CR values of the remaining 40 questions all reached the significant level of 0.001. Correlation analysis was performed between the scores of each question and the total questionnaire score. The analysis results show that the r value of Q17, Q18, and Q33 questions is lower than 0.2 (20). Therefore, according to the project analysis results, questions Q17, Q18, and Q33 are deleted. There are 38 questions left in the end.

**Table 2:** Questions and descriptive statistics of CSPLQ (*N* = 501).

**Coding**	**Project description**	***Mean***	***SD***	**Skewness**	**peak**	**CR**	**Correlation coefficient with total scale**
Q1	I can walk, run, jump, throw, hit, kick, etc.	4.269	0.980	1.999	−1.507	−9.310	0.438^**^
Q2	I can do a standard plank	3.659	1.119	−0.509	−0.500	−18.583	0.659^**^
Q3	I am good at coordinated movements	3.633	0.947	0.037	−0.499	−17.257	0.670^**^
Q4	I can do those movements that require high precision very well	3.232	0.965	−0.300	−0.128	−19.527	0.709^**^
Q5	I have good hand-eye coordination	3.615	0.877	−0.085	−0.309	−16.193	0.661^**^
Q6	My body has a better sense of rhythm than my peers	3.244	0.921	−0.140	−0.070	−16.540	0.685^**^
Q7	I'm noticeably faster than my peers	3.098	0.980	−0.217	−0.082	−16.840	0.664^**^
Q8	I did better than others in strength tests	3.030	0.911	−0.082	0.084	−16.163	0.640^**^
Q9	I am good at endurance activities such as cycling, running, swimming, etc.	3.024	1.039	−0.485	0.070	−14.284	0.606^^**^^
Q10	I am more flexible than most of my peers	3.020	1.062	−0.573	−0.030	−10.907	0.498^**^
Q11	I can do activities that require flexibility	3.082	0.955	−0.233	0.044	−15.818	0.658^**^
Q12	I have good balance	3.357	0.884	0.165	−0.241	−16.141	0.670^**^
Q13	I'm better at sports than most of my friends	3.128	1.008	−0.395	−0.117	−18.016	0.692^**^
Q14	Most sports are easy for me	3.192	1.006	−0.326	−0.154	−21.589	0.759^**^
Q15	I do physical exercise or other physical activity almost every day	3.481	0.939	−0.158	−0.279	−14.750	0.596^**^
Q16	I do physical activity lasting 30 min or more at least 3 times a week (e.g., cycling, running, playing, etc.)	3.645	1.063	−0.692	−0.337	−15.908	0.570^**^
Q17	Almost every day I spend a lot of time on my phone or on my computer	2.643	0.933	0.037	0.292	−1.737	0.109*
Q18	I spend most of my waking hours sitting or lying down	2.733	0.968	−0.150	0.211	−2.875	0.152^**^
Q19	I know the knowledge of a reasonable diet	3.337	0.897	−0.108	−0.114	−11.223	0.493^**^
Q20	I know the standard for good sleep	3.615	0.877	0.053	−0.416	−11.098	0.496^**^
Q21	I know what healthy habits are	3.776	0.786	0.042	−0.327	−12.246	0.545^**^
Q22	I know that health includes physical, mental, social adaptation and moral health	3.856	0.819	−0.246	−0.299	−11.369	0.528^**^
Q23	I have mastered basic physical exercise knowledge	3.499	0.841	−0.094	−0.139	−17.997	0.676^**^
Q24	I have mastered the knowledge of sports safety protection	3.535	0.808	−0.248	−0.057	−17.939	0.681^**^
Q25	I know the WHO recommended physical activity guidelines for my age group	2.926	1.059	−0.596	0.056	−12.894	0.558^**^
Q26	I know the World Health Organization recommended guidelines for sedentary behavior for my age group	2.900	1.104	−0.736	0.055	−12.207	0.528^**^
Q27	I know that physical activity has many benefits for the human body	4.144	0.836	−0.267	−0.646	−9.981	0.463^**^
Q28	I think being physically active brings me joy	3.926	0.863	−0.847	−0.214	−19.359	0.675^**^
Q29	I think being physically active will keep me in better shape	4.160	0.838	0.051	−0.738	−12.545	0.517^**^
Q30	I think participating in physical activity increases my social interaction	3.874	0.866	−0.947	−0.144	−19.435	0.654^**^
Q31	I think being physically active makes me healthier	4.259	0.759	−0.573	−0.611	−10.522	0.464^**^
Q32	I need to increase physical activity to improve my physical test scores	3.994	0.840	−0.330	−0.436	−10.411	0.471^**^
Q33	Physical exercise is mandatory in schools	4.030	1.051	0.186	−0.910	−3.342	0.144^**^
Q34	My friends regularly engage in physical activity (e.g., cycling, running, playing ball, etc.)	3.709	0.973	−0.387	−0.370	−15.101	0.565^**^
Q35	My parents often push me to do physical activities (like biking, running, playing ball, etc.)	3.339	1.077	−0.363	−0.341	−9.848	0.445^**^
Q36	I enjoy watching various sports (games)	3.327	1.125	−0.569	−0.213	−16.275	0.618^**^
Q37	I think people who are in good shape are more attractive	3.900	0.952	0.213	−0.680	−8.946	0.421^**^
Q38	I think people in physical activity are very dynamic	4.146	0.795	0.124	−0.650	−9.481	0.440^**^
Q39	I am satisfied with my level of physical function	3.259	0.994	−0.272	−0.220	−16.292	0.632^**^
Q40	I am confident in my physical mobility	3.415	1.021	−0.313	−0.323	−19.964	0.716^**^
Q41	My body is more attractive than my peers	3.138	1.067	−0.582	−0.009	−16.059	0.649^**^

*represents a significance level of 1%.

** *P < 0.01*.

##### Exploratory Factor Analysis

In order to analyze the structure of CSPLQ, SPSS 17.0 software was used to conduct exploratory factor analysis on 501 pre-tested survey data. The results showed that the Bartlett sphericity test was ~4,991.83 chi-square, and the KMO value was 0.943, reaching the significant level of 0.001, indicating that the new questionnaire is suitable for factor analysis. The data in this study were rotated using the maximum variance rotation method (Varimax). Combined with variance contribution rate and gravel plot analysis, seven factors are obtained, and the eigenvalues are all >1. The variance explanation rates of these seven factors after rotation are 16.142, 13.681, 8.049, 7.922, 6.919, 6.199, and 5.793%, respectively. The cumulative variance explained rate after rotation is 64.704%. Since the Q17, Q18, and Q33 questions have been deleted during the project analysis, there are 7 secondary indicators remaining. Therefore, the total number of items in this exploratory factor analysis is 38. The seven factors extracted by factor analysis are consistent with the original dimension concept, and the analysis results are shown in Table 3. It can be seen from the table that the factor loadings of all the questionnaire items on their respective factors are >0.40. It shows that the questionnaire has good construct validity. Factor 1 named motor skills, factor 2 named motivation to participate in physical activity, factor 3 named motion skills, factor 4 named confidence to participate in physical activity, factor 5 named cognition of physical activity, factor 6 named physical activity, and factor 7 named cognition of healthy lifestyle. According to the results of factor analysis, the Cronbach α coefficients among the items of each factor were tested. The Cronbach α coefficients of motor skills, motion skills, physical activity, cognition of healthy lifestyle, cognition of physical activity, motivation to participate in physical activity, and confidence to participate in physical activity were 0.867, 0.913, 0.765, 0.768, 0.833, 0.857, and 0.829, respectively. Since there is only one dimension of physical activity remaining in the behavioral field, after consulting experts, the physical activity dimension, motor skills, and motion skills dimensions are combined to form the physical and behavioral field. Therefore, the final CSPLQ is 3 fields and 7 dimensions in total 38 The structure of the assessment for each question.

**Table 3:** CSPLQ exploratory factor analysis results (*N* = 501).

**Coding**	**Factor loadings**							**Commonality**
	**Factor 1**	**Factor 2**	**Factor 3**	**Factor 4**	**Factor 5**	**Factor 6**	**Factor 7**	
Q1			0.693					0.552
Q2			0.637					0.681
Q3			0.710					0.770
Q4			0.600					0.743
Q5			0.633					0.698
Q6	0.674							0.714
Q7	0.646							0.613
Q8	0.604							0.557
Q9	0.619							0.556
Q10	0.740							0.618
Q11	0.816							0.751
Q12	0.705							0.636
Q13	0.606							0.677
Q14	0.629							0.703
Q15						0.670		0.660
Q16						0.698		0.654
Q19							0.671	0.655
Q20							0.725	0.668
Q21							0.690	0.709
Q22							0.427	0.584
Q23					0.593			0.665
Q24					0.571			0.654
Q25					0.729			0.751
Q26					0.742			0.770
Q27		0.713						0.574
Q28		0.665						0.652
Q29		0.809						0.689
Q30		0.642						0.622
Q31		0.821						0.693
Q32		0.686						0.534
Q34		0.467						0.564
Q35		0.617						0.517
Q36		0.486						0.543
Q37		0.451						0.521
Q38		0.664						0.620
Q39				0.692				0.685
Q40				0.643				0.717
Q41				0.575				0.621

##### Confirmatory Factor Analysis

To verify the stability of the content structure of the CSPLQ, this study used AMOS 23.0 to test Group 2.The evaluation model uses 38 items of CSPLQ as significant variables. Seven first-order factor latent variables (motor skills, motion skills, physical activity, cognition of healthy lifestyle, cognition of physical activity, motivation to participate in physical activity, and confidence to participate in physical activity) were respectively, formed. Among them, three first-order factors of motor skills, motion skills and physical activity constitute a second-order latent variable (physical and behavior domain). Two first-order factors, healthy lifestyle cognition and physical activity cognition, formed a second-order latent variable (cognitive domain). Two first-order factors of motivation to participate in physical activity and confidence to participate in physical activity constitute a latent variable (emotional domain) of a second-order factor. The analysis of the validation factor was performed using the maximum likelihood method.

**Figure 2:**
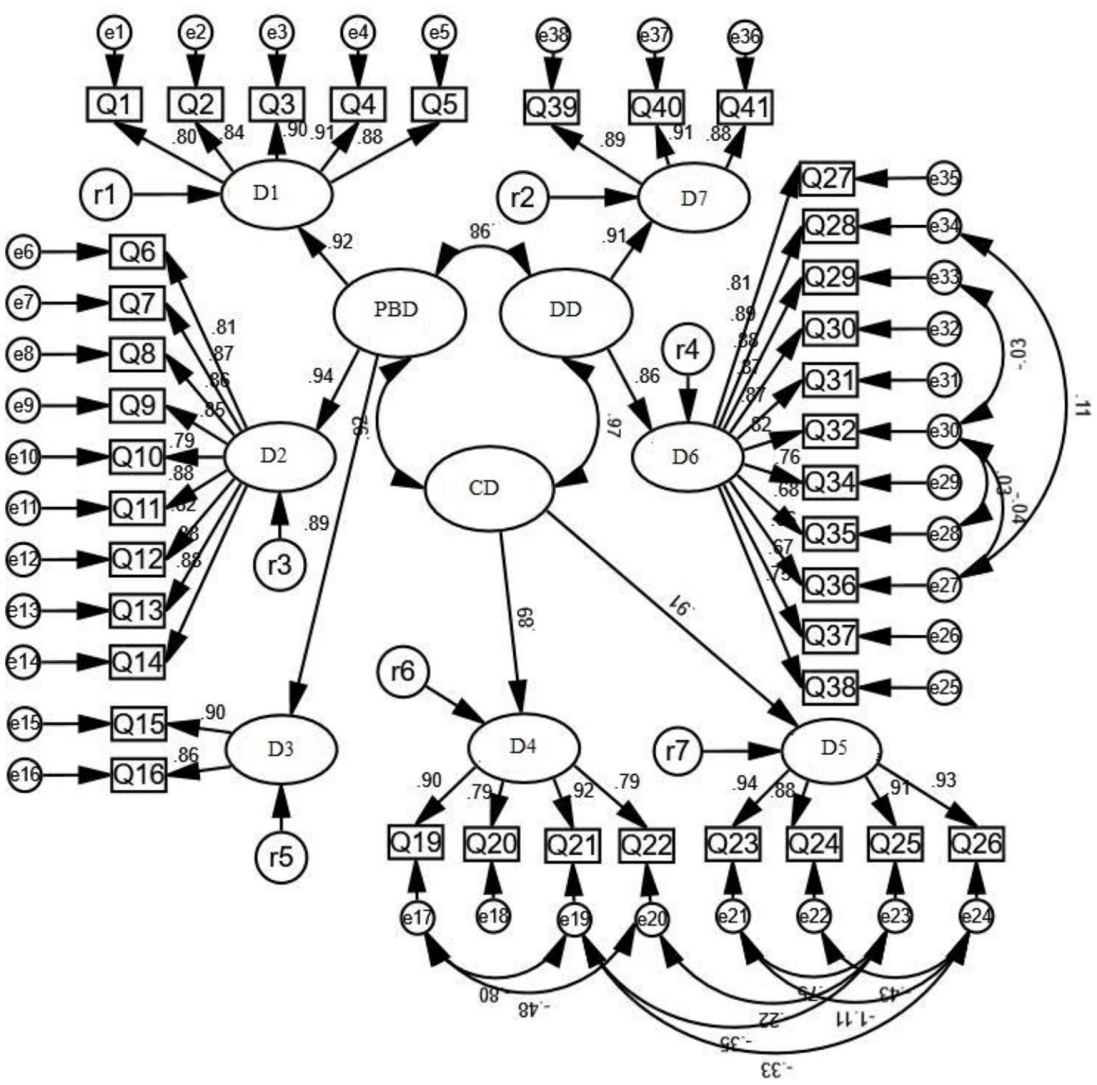
The measurement model of CSPLQ(*N* = 1,173). D1, Motor skills; D2, Motion skills; D3, Physical activity; D4, Cognition of physical activity; D5, Cognition of healthy lifestyle; D6, Motivation to participate in physical activity; D7, Confidence to participate in physical activity; PBD, Physical and behavioral domain; CD, Cognitive domain; Emotional domain.

Confirmatory factor analysis of CSPLQ was carried out using 1,173 survey data of formal test. Table 4 shows the fitting indexes of the original model and the revised model. The results of data analysis showed that the revised final model had good construct validity (see Figure 2). The factor loadings of all item bars are higher than 0.7, indicating that each factor has good convergent validity.

**Table 4:** The results of the second-order confirmatory factor analysis of the CSPLQ.

	***X^2^/df***	**TLI**	**CFI**	**RMSEA**
Initial model	4.28	0.823	0.837	0.070
Corrected model	3.07	0.901	0.911	0.062

##### Reliability Analysis

SPSS 22.0 software was used for statistical analysis of the Cronbach α coefficients of the three sub-question tables and the total questionnaire. The Cronbach α coefficients were 0.936, 0.900, 0.915, and 0.961, respectively.

*Relationship With Other Variables*. This study also looked at the relationship between college students' physical literacy and other variables, including athletic ability, physical condition, physical attractiveness (21), physical fitness, frequency of physical activity (average weekly voluntary physical activity frequency in the past month), and physical activity duration (every duration of autonomous physical activity). The inspection results showed that the correlation coefficients between the dimensions and total scores of college students' physical literacy and these variables reached a significant level of *P* < 0.05 (Table 5).

**Table 5:** Correlation analysis of CSPLQ with other variables (*N* = 1,173).

**Dimension**	**Exercise ability**	**Physical condition**	**Physical attractiveness**	**Physical quality**	**Frequency of physical exercise**	**Duration of physical exercise**
Motor skills	0.398^**^	0.400^**^	0.279^**^	0.379^**^	0.178^**^	0.241^**^
Motion skills	0.594^**^	0.559^**^	0.446^**^	0.597^**^	0.192^**^	0.290^**^
Physical activity	0.568^**^	0.613^**^	0.406^**^	0.552^**^	0.429^**^	0.342^**^
Cognition of physical activity	0.137^**^	0.154^**^	0.117^**^	0.156^**^	0.199^**^	0.198^**^
Cognition of healthy lifestyle	0.390^**^	0.418^**^	0.331^**^	0.395^**^	0.116^**^	0.213^**^
Motivation to participate in physical activity	0.390^**^	0.476^**^	0.296^**^	0.388^**^	0.257^**^	0.248^**^
Confidence to participate in physical activity	0.449^**^	0.482^**^	0.388^**^	0.434^**^	0.180^**^	0.262^**^
Physical literacy	0.538^**^	0.570^**^	0.413^**^	0.534^**^	0.267^**^	0.322^**^

** *P < 0.01*.

## Discussion

This paper provides evidence for the validity of the CSPLQ. Evidence of content validity is provided for all processes from defining the domain, constructing definitions, generating items for expert review and response processes, content structure analysis, and relationships with other variables (25). For item generation, we referred to existing physical literacy assessment tools because they have good comprehension and a good classification of indicators. However, we found some differences in the structure of the assessment models between the different tools. For example, Australia's structural model of physical literacy assessment consists of four domains: physical, mental, social and cognitive (10). The physical literacy measure developed by the Canadian Care for Life project has five physical, behavioral, cognitive, psychological and social dimensions (26). The physical literacy assessment tool developed by the Canadian Lifetime Sport Program is divided into a professional, coaching, parent and self-test version and contains four main dimensions: physical, behavioral, psychological and cognitive (9). The Canadian Healthy Active Living and Obesity Research Group designed a physical literacy assessment tool that includes physical, behavioral, psychological and cognitive dimensions (9), and Allan constructed a physical literacy assessment tool that focuses on athletes and includes physical, behavioral, cognitive, psychological and social dimensions. In order to obtain more agreement from Chinese scholars, we chose Li 's division of the physical literacy structure, which is currently more agreed by Chinese scholars in this field, and divided the dimensions of physical literacy measurement into four domains: emotional, physical, cognitive and behavioral domains. Most of the experts involved in this study had knowledge related to sport measurement and evaluation or physical literacy research, which was a strength of our study, but given that our study was an initial exploratory study aimed at designing a validated self-report questionnaire on physical literacy, our team endeavored to describe our objectives and methods to the experts in order to provide them with a deeper understanding of the assessment of physical literacy among university students.

We designed a forty-three-item preliminary questionnaire based on the literature and recommendations from a three-person expert panel. The experts were asked to rate and make suggestions on the relevance, clarity and comprehensiveness of the questionnaire items. After this process, two observation points of “daily lying time” and “frequency of physical activity in a week” have been deleted, and two observation points have been modified, such as “body shape preference” being changed to “aesthetic preference,” and “activity participation” being changed to “activity” appreciation.” Since this process leaves only two entries in both dimensions of our behavioral domain, we naturally expect that it might be less effective later on. But considering that we can also continue to judge their effectiveness through content structure analysis, we keep these dimensions and entries. During the response process, the college students we invited helped to revise the description of the questionnaire, so that our question and answer description methods were more in line with the language habits and acceptance methods of college students. After evaluation, the description of Q24 “I have mastered the knowledge of sports safety protection” was changed to “I have mastered the knowledge of sports safety protection,” and the Q36 “I like watching various sports events” was changed to “I like watching various sports activities very much (competition).”

To verify the stability of this structure, we validated it in another sample of university students. The results of the model fit showed that the content structure of the CSPLQ was relatively stable. As there was no Mandarin version of the College Student Physical Literacy Questionnaire for us to make reference to the relationship between the relevant variables, we chose some variables from the Physical Esteem Scale and Physical Activity Behavior to observe the relationship between physical literacy and them. The results showed that the correlation coefficients between physical literacy and other variables, including athletic ability, physical condition, physical attractiveness, physical fitness, frequency of physical activity and length of physical activity among university students, reached a significant level of *P* < 0.05. This indicates that our questionnaire may be effective in assessing the physical literacy of college students. However, we do not suggest weighted scores for the final scale, and we think this is a question that needs to be further investigated in follow-up studies. Although this study proposes a valid college student self-reported questionnaire to identify college students' physical literacy, there are still some limitations and weaknesses that can be considered for future research. For example, although almost all of our experts know something about physical literacy, none of them has actually done research on physical literacy assessment, so the authority of our experts may affect the validity of our questionnaire. At the same time, we have only two items in one dimension. Although their validity has been verified by other samples, we hope to expand the evaluation items of this dimension in future research. And our sample size is relatively small, and the research sample can be further expanded in the future. Future research could also add test-retest checks to increase the reliability of the questionnaire. Finally, the strength of our research is to open up a new method to objectify the physical literacy of college students, which is a valid self-report questionnaire of college students to identify their physical literacy. Therefore, our study is the first step in developing a standard questionnaire.

## Conclusion

The CSPLQ has sufficient validity evidence. The development of this tool shows that this tool has validity evidence for its content, response process, internal structure and relationship with other variables.

## Data Availability Statement

The raw data supporting the conclusions of this article will be made available by the authors, without undue reservation.

## Ethics Statement

The studies involving human participants were reviewed and approved by the Ethics Review and Approval of the Academic Committee of the Physical Education College of Guizhou Normal University (No. 20210310). The patients/participants provided their written informed consent to participate in this study.

## Author Contributions

LL conceived the study and performed the data analysis and interpretation. LL and NS prepared the manuscript. JYU, CL, LZO, LZA, SL, XG, and JYA were involved in data collection. JH and XZ were involved in the revision and guidance of the paper. All authors have read and approved the final manuscript.

## Funding

This research was funded from the East China Normal University-Zhongxu Postdoctoral Workstation Fund (No. 2019001), the Guizhou Provincial Department of Education Youth Growth Project Fund (Qianjiao He KY [2021] 291), the Guizhou Province Education Planning Fund Project (2021A058), and Guizhou Normal University Teaching Content and Curriculum System Reform Project ([2021]xjg, No. 03).

## Conflict of Interest

The authors declare that the research was conducted in the absence of any commercial or financial relationships that could be construed as a potential conflict of interest.

## Publisher's Note

All claims expressed in this article are solely those of the authors and do not necessarily represent those of their affiliated organizations, or those of the publisher, the editors and the reviewers. Any product that may be evaluated in this article, or claim that may be made by its manufacturer, is not guaranteed or endorsed by the publisher.
